# Shoulder Dystocia: A Comprehensive Literature Review on Diagnosis, Prevention, Complications, Prognosis, and Management

**DOI:** 10.3390/jpm14060586

**Published:** 2024-05-30

**Authors:** Panagiotis Tsikouras, Sonia Kotanidou, Konstantinos Nikolettos, Nektaria Kritsotaki, Anastasia Bothou, Sotiris Andreou, Theopi Nalmpanti, Kyriaki Chalkia, Vlassios Spanakis, Panagiotis Peitsidis, George Iatrakis, Nikolaos Nikolettos

**Affiliations:** 1 Department of Obstetrics and Gynecology, Democritus University of Thrace, Dragana, 681 00 Alexandroupolis, Greece; kotanidou.so@gmail.com (S.K.); k.nikolettos@yahoo.gr (K.N.); nektaria.97@hotmail.com (N.K.); soterisand@hotmail.com (S.A.); theonalmpanti@hotmail.com (T.N.); kikichalkia@yahoo.gr (K.C.); spanakvls@outlook.com.gr (V.S.); nnikolet@med.duth.gr (N.N.); 2 Midwifery Department of Neonatology, University Hospital Alexandra, Vasilissis Sofias Ave. 80, 115 28 Athens, Greece; natashabothou@windowslive.com; 3 Department of Obstetrics and Gynecology, Fetal Medicine Hospital Helena Venizelou, Elenas Venizelou 2, 115 21 Athens, Greece; peitsidiobgyn@gmail.com; 4 Department of Midwifery, University of West Attica, Agiou Spyridonos 28, 122 43 Egaleo, Greece; giatrakis@uniwa.gr

**Keywords:** shoulder dystocia, diagnosis, complications, prevention

## Abstract

The term dystocia refers to labor characterized by a slow progression with delayed rates or even pauses in the dilation of the cervix or the descent of the fetus. Dystocia describes the deviation from the limits that define a normal birth and is often used as a synonym for the term pathological birth. Shoulder dystocia, also known as the manual exit of the shoulders during vaginal delivery on cephalic presentation, is defined as the “failure of the shoulders to spontaneously traverse the pelvis after delivery of the fetal head”. This means that obstetric interventions are necessary to deliver the fetus’s body after the head has been delivered, as gentle traction has failed. Abnormal labor (dystocia) is expressed and represented in partograms or by the prolongation of the latent phase or by slowing and pausing in the phases of cervical dilatation and fetal descent. While partograms are helpful in visualizing the progress of labor, regular use of them has not been shown to enhance obstetric outcomes considerably, and no partogram has been shown to be superior to others in comparative trials. Dystocia can, therefore, appear in any phase of the evolution of childbirth, so it is necessary to simultaneously assess all the factors that may contribute to its abnormal evolution, that is, the forces exerted, the weight, the shape, the presentation and position of the fetus, the integrity and morphology of the pelvis, and its relation to the fetus. When this complication occurs, it can result in an increased incidence of maternal morbidity, as well as an increased incidence of neonatal morbidity and mortality. Although several risk factors are associated with shoulder dystocia, it has proven impossible to recognize individual cases of shoulder dystocia in practice before they occur during labor. Various guidelines have been published for the management of shoulder dystocia, with the primary goal of educating the obstetrician and midwife on the importance of a preplanned sequence of maneuvers, thereby reducing maternal and neonatal morbidity and mortality.

## 1. Introduction

The concept of shoulder dystocia has been documented for over two centuries. It is characterized by the anterior shoulder becoming wedged behind the pubic symphysis following the birth of the fetal head. This situation arises when the biacromial diameter exceeds the pelvic diameter. Shoulder dystocia is identified by the requirement for further obstetric interventions to facilitate the delivery of the fetal shoulders during vaginal childbirth, as standard gentle downward traction applied to the head proves insufficient to deliver the anterior shoulder [1,2].

Shoulder dystocia arises when the anterior shoulder becomes trapped beneath the pubic symphysis or, less commonly, the posterior shoulder at the pubic iliac crest. The “turtle point sign” is characterized by the retraction of the fetal head after childbirth towards the perineum following childbirth. Typically, after the delivery of the head, the arm is suffocatingly pressed against the pregnant woman and encounters resistance as the anterior shoulder becomes lodged behind the symphysis pubis [3,4]. 

Shoulder dystocia comprises 0.15% to 1.7% of all deliveries, though this frequency fluctuates depending on the fetal birth weight [3,4,5,6]. Hence, it manifests in approximately 0.3% to 1% of neonates weighing between 2.5 and 4 kg at birth, escalating to 5% to 7% in neonates weighing between 4 and 4.5 kg [7,8,9,10]. More than half of shoulder dystocia cases involve fetuses with a normal birth weight, rendering it an unpredictable complication [3,11,12,13]. There is evidence that the incidence of shoulder dystocia has increased over time, due to the increasing birth weight of newborns, and is an obstetric emergency. Furthermore, considering that the engagement of the fetal head is a determinant element when deciding on operative vaginal delivery, a transperineal ultrasound with a transvaginal digital examination can add to the avoidance of the deleterious failure of operative vaginal delivery [14].

## 2. Diagnosis 

In instances where antenatal and perinatal factors are linked to the potential occurrence of shoulder dystocia, it is imperative for all maternity personnel to promptly identify early indicators of this condition, such as the failure of the shoulders to descend. Looking at it objectively, shoulder dystocia is defined as the extension of the time interval between the delivery of the head and the delivery of the shoulders beyond 60 s. A minority of shoulder dystocia deliveries may exhibit the “turtle sign,” characterized by the retraction of the baby’s head, reminiscent of a turtle retracting into its shell, accompanied by a red, swollen face. This phenomenon occurs when the baby’s shoulder becomes obstructed by the maternal pelvis. Dystocia refers to the departure from the parameters that delineate a typical birth process, and, hence, the term “pathological birth” is frequently employed synonymously. In challenging deliveries, there is a discernible sluggish advancement marked by delayed intervals or pauses in cervical dilatation or the descent of the fetus.

Dystocia, depending on their cause, is divided into three major categories:

(a) Dystocia that comes from the insufficiency of the forces exerted during childbirth, both by the uterus and by the placenta [15], and aimed at the exit of the fetus. They include the primary or secondary dysfunction of the uterus during the first and second stages of labor and the failure of voluntary expulsive efforts of the epitome during the second stage of labor, respectively.
(b) Dystocia attributed to fetal factors. They include abnormal shapes, presentations, positions, large fetuses, and congenital anomalies of the fetus.
(c) Dystocia that stems from congenital or acquired abnormalities affecting the pelvic canal. These anomalies encompass not only structural irregularities of the bony pelvis but also abnormalities related to the soft tissues that contribute to the composition of the pelvic canal [15,16]. The above distinction of dystocia is not absolute, as, in many cases, causes belonging to different categories contribute to the manifestation of dystocia. For example, a narrow pelvis creates the conditions for the appearance of an abnormal shape, presentation, and position of the fetus [17].

### 2.1. Dystocia Caused by Maternal Factors

It has been estimated that contractions of at least 15 mmHg are required to achieve cervical dilation, while normal uterine contractions usually have an intensity of 60 mmHg [18]. 

We distinguish uterine dysfunctions into two categories:

(a) Hypotonic dysfunction. Resting basal tone is low, and contractions, although synchronized, have little intensity, failing to achieve cervical dilatation.
(b) Hypertonic or unco-ordinated dysfunction.

In cases of hypertonic dysfunction, the fundamental resting tone of the uterus is elevated, leading to frequent and prolonged contractions that are ineffective in terms of cervical dilation. Specifically, the lower and middle sections of the myometrium contract more forcefully than the upper portion, and/or there is a lack of synchronization in the impulses originating from the two horns of the uterus. This lack of synchronization inhibits the propagation of contractions as a cohesive downward wave required for achieving dilation.

Epidural anesthesia, chorioamnionitis, and maternal positioning during labor have all been identified as potential contributors to uterine dysfunction. While conflicting viewpoints exist, evidence suggests that mobilization during labor, when not contraindicated, does not pose significant complications for primigravidas. In fact, it is often welcomed by expectant mothers, as it can have a positive impact on their psychological well-being, empowering them with the strength to persevere through childbirth [3,5,13].

During the second stage of labor, the descent and delivery of the fetus through the pelvic canal to the vulva are facilitated by both the forces exerted by the uterus and the voluntary expulsive efforts of the mother. The latter is generated by the contraction of the abdominal muscles and can be quite forceful, reaching pressures of 100–150 mmHg [19]. Their role is considered catalytic and their deficiency can lead to dystocia.

The causes that can lead to a deficiency of the extrusive forces of the healthy parturient are heavy sedation, regional anesthesia, and the voluntary suspension of the extrusive effort due to the intense pain it causes. In other instances, the involvement of the parturient experiencing neuromuscular disorders may be deemed minimal, whereas, in cases of heart disease, there typically exists a relative contraindication [19]. In such scenarios, delivery may be facilitated through the use of forceps, vacuum extraction, or caesarean section.

### 2.2. Dystocia Attributed to Fetal Factors

Dystocia attributed to fetal factors include abnormal shapes, presentations, positions, a large fetus, and congenital anomalies of the fetus.

#### 2.2.1. Abnormal Shapes and Presentations

In roughly 97% of pregnancies, the presentation during delivery is cephalic, whereas, in 3%, it is breech. In less than 0.5% of cases, the fetus assumes a transverse or oblique position.

(a) Occipito-posterior fetal position

In such a position, the occiput of the fetus, specifically the posterior fontanelle which serves as its guiding point, is situated within one of the posterior quadrants of the pelvis, typically the right quadrant. It is observed that the right posterior position occurs approximately five times more frequently than the corresponding left position. It is an unstable-transitional position, since, in most cases (90%), it is directed to the anterior occipital position [7]. It is the most frequent cause of delay in the progress of childbirth [7]. The construction of the pelvis (narrow, android, or flattened pelvis), the right deviation of the uterus due to the pressure of the sigmoid, the anterior placenta, and the relaxation of the abdominal walls of the pregnant woman are predisposing factors for the occurrence of the above-mentioned abnormal fetal head presentation [20,21]. Diagnosing the right posterior position is facilitated not only by a vaginal examination but also by the induration often palpated above the pubic symphysis, the increased audibility of fetal heartbeats across a wider area of the abdomen especially after membrane rupture, and the noted delay in the progression of labor [7,20,21]. At the same time, the parturient shows intense back pain, as well as a tendency to push out before the dilation is complete, apparently from the pressure exerted by the head on the intestine and the sacrum. Approximately 70% of the time, labor progresses with the head bending perfectly and a significant rotation of 135° into the occipito-anterior position, while, in the remaining 30%, labor occurs with incomplete head bending and a smaller rotation of 45° into the occipito-posterior position [7,20,21].

(b) Occipital transverse position

In this position, the occiput of the fetus, particularly the posterior fontanelle which serves as its guiding point, is situated in a transverse position, either to the right or to the left. It is considered an unstable-transitional projection, since, most of the time, it turns to an anterior occipital position, although it is usually maintained until the descent of the head into the birth canal [7,20,21]. It is attributed to the shape of the pelvis (flat pelvis) and the secondary inactivity of the uterus. When the cause is the dysfunction of the uterus, we strengthen the contractions with oxytocin, while, when the time limits of the extrusion stage are exhausted, the solution is given by the embryo wound (metal or suction) or caesarean section depending on the height of the projecting degree [7,20,21].

(c) Parietal presentation

In this position, the fetus’s head protrudes with the parietal bones, forming the dome of the skull. The prominent anterior fontanelle, which acts as its guiding point, is palpated at the center of the pelvis. Moreover, the head is neither flexed nor extended but is rather in a neutral position.

This is an unstable-transitional position, since it often passes into an occipital or, more rarely, into a frontal and personal position. If the labor does not progress and the position remains parietal, delivery via caesarean section is preferred. It is important to emphasize that the use of vacuum extraction is not recommended, as applying vacuum in this position should be directed towards the anterior fontanelle, which is not feasible in this scenario.

(d) Brow presentation

This presentation, characterized by the fetus presenting with the forehead and the nasion serving as the guiding point, is exceedingly rare. It is classified as an unstable-transitional presentation, often transitioning into either a face or parietal presentation. The appearance of this presentation is attributed to factors such as polyhydramnios, a narrow or deformed pelvis, and prematurity. In most cases, labor progress is delayed or becomes impossible, necessitating delivery via caesarean section. The historical practice of attempting to convert a brow presentation into an occipital position through various manipulations is no longer considered appropriate in modern obstetrics [7,16,17,18,19].

(e) Face presentation

In this presentation, the fetus presents with the face as the leading part. The presenting part is mentum. Depending on the position of the eye, which serves as the guiding point in this type of presentation, it is classified as either anterior or posterior. During the vaginal examination, this presentation can pose a diagnostic challenge as it may be mistaken for a frank breech presentation. This confusion can arise because the mouth may be misidentified as the anus, and the ischial spines may be mistaken for the zygomatic bones [3]. Among the causes are an elongated neck of the fetus or the umbilical cord coiling, which can result in the extension of the head. Additionally, predisposing factors include the presence of anencephaly-acrania, fetal macrosomia, multiparity, and a narrow pelvis.

The presentation of the fetus plays a crucial role in its delivery process: in an anterior face presentation, vaginal delivery can often proceed spontaneously, whereas, in a posterior presentation, a caesarean section will likely be necessary. In practice, regardless of the presentation, caesarean section is often the chosen approach for face presentation deliveries.

(f) Occipito-posterior positions

In this presentation, the fetal head becomes wedged with the sagittal diameter aligned with the anteroposterior diameter of the pelvic entrance, either in an occipito-pubic or occipito-sacral position. Diagnosis is typically confirmed through a vaginal examination. The resolution of dystocia through head turning rarely occurs. While the careful administration of oxytocin in cases of insufficient labor may aid in facilitating delivery, more often than not, caesarean section becomes the preferred method of delivery.

(g) Uneven cephalic presentation
  The consolidation of the fetal head, indicating its passage into the pelvic inlet where the biparietal diameter has surpassed the level of entry, can be achieved through three methods:
  (1) The two parietal bones should be at the same height; i.e., the sagittal suture should be exactly in the middle of the pelvis. This presentation is characterized as an isocline.
  (2) The posterior parietal bone should be at a lower level, i.e., project, so that the sagittal suture is closer to the anterior wall of the pelvis. This presentation is characterized as posterior asynclitism.
  (3) The anterior parietal bone should be at a lower level, i.e., project, so that the sagittal suture is closer to the posterior wall of the pelvis. This presentation is characterized as anterior asynclitism. The uneven positions of the cephalic presentation are usually presented in a flattened pelvis and are a serious cause of disproportion and dystocia and, most often, lead to a caesarean section [20].

#### 2.2.2. Large or Overweight Fetus

Typically, the threshold for diagnosing macrosomia is a birth weight between 4000 and 4500 g, or higher. However, achieving a universal agreement on this definition can be challenging due to variations in clinical practice and regional differences. About 10% of newborns are large at birth and 2% are extremely large. Predisposing factors include obesity (>70 kg), diabetes mellitus, maternal weight gain greater than expected for pregnancy (>20 kg), multiparity, prolonged pregnancy, and the history of previous delivery of a large fetus [7,18,19,20,21].

It is estimated that 10% of large fetuses will develop shoulder dystocia. For this reason, the American College of Obstetricians and Gynecologists in 2002 issued a guideline for performing a caesarean section for fetuses with an estimated birth weight greater than 5000 g in non-diabetic and 4500 g in diabetic pregnant women [7,18,19,20,21].

### 2.3. Dystocia Arising from Congenital or Acquired Anomalies of the Pelvic Canal 

They include the anomalies of the bony pelvis and the soft tissues, which participate in the structure of the pelvic canal [22,23,24].

(a) Abnormalities of the bony pelvis
  Radiological studies by Caldwell and Moloy, in the first half of the 20th century, led to the classification of the female pelvis into four types:
  (1) The female type pelvis (41.4%) is characterized by a round or slightly oval entrance with large diameters between the ischial spines and the ischial tuberosities. Women with this type of pelvis typically have a girth span greater than their shoulder span. This type of female pelvis is not typically associated with dystocia problems.
  (2) The android type (32.5%) is characterized by a triangular-wedge-shaped entrance with the apex at the pubic symphysis and a shorter diameter between the ischial spines. Women with this type of pelvis have the same opening in the circumference and shoulders.
  (3) The humanoid type (23.5%) is characterized by an oval entrance with an anteroposterior diameter greater than the transverse one. It looks like the pelvis of great apes.
  (4) The flat-bellied type (2.6%) is characterized by an oval entrance with a transverse diameter greater than the anterior one.

The last three types often present dystocia problems. According to Cadwell’s observations on the Hellenes, the distribution of the aforementioned types is as follows: gynoid (72%), android (23.7%), anthropoid (3%), and platypyeloid (1.3%). Mixed types of female pelvis, combining characteristics of these types, are frequently observed, and significant racial differences have been noted [22,23,24].

However, apart from the type of pelvis, it is possible to have acquired abnormalities in its formation, most of the time as a result of the fracture of the innominate bones after traffic accidents. In the past, pathological conditions of the bones, such as rickets, were a frequent cause of a narrow and deformed pelvis.

The diagnosis of a narrow pelvis with a reduction in the diameters of the inlet, the vasculature, the stricture, and the outlet can be carried out with clinical pyelometry, radiological control, and pyelometry with axial or magnetic tomography. Many times, however, the assessment is difficult, with the result that the pelvis must be “tried in labor” before the definitive diagnosis is made.

(b) Abnormalities of the soft tissues

Abnormalities of the soft tissues involved in the structure of the pelvic canal can be causes of dystocia. Among them are isthmic fibroids of the uterus, appendage tumors, colon tumors, and bladder distention.

## 3. Frequency of Dystocia

In contemporary discussions, cephalopelvic disproportion is frequently mentioned as a determining factor necessitating the termination of labor through a caesarean section. The term cephalopelvic disproportion was used as early as the 19th century to describe the disproportion between the diameters of the fetal head and the pelvic canal, through which it will have to pass during delivery.

It accurately depicted the instances of pregnant women afflicted with rickets, leading to a constricted pelvis, which affected up to 15% of cases in certain regions at the outset of the 20th century. However, such severe cephalopelvic disproportion, rendering childbirth progression impossible, is now uncommon, with the term often misapplied in contemporary usage.

Most of the time, it is a relative disproportion, in which the delivery can be completed with great difficulty and usually with some obstetric intervention [7,25,26]. Excluding cases of absolute disproportion, which can be diagnosed clinically and through ultrasound, most instances of dystocia are identified during or after labor.

Dystocia is the most common indication for a first caesarean section. As a history of caesarean section often leads to subsequent delivery by caesarean section, it has been estimated that dystocia accounts for approximately 50–60% of caesarean sections performed in the US [7,25,26]. In general, the rate of dystocia in primipara is less than 10% [7,25,26].

Recently, there has been a suggestion that the observed rise in the rate of dystocia today may be attributed to the rapid changes in environmental conditions and lifestyle outpacing the pace of evolution, as defined by Darwin’s theory of natural selection in physics [27,28]. 

## 4. Pathophysiology of Shoulder Dystocia

In the time interval between the birth of the head and the body of the fetus (head-to-body delivery time), the fetus develops acidosis, which can lead to hypoxia. Fetal acidosis can result from various factors, including the compression of the umbilical cord or neck vessels, congestion in the fetal venous network, the stimulation of the brain’s vagus nerve leading to bradycardia, or a combination of these elements. The regulation of fetal cardiac function is primarily governed by the autonomic nervous system, with minimal influence from the somatic nervous system.

Fluctuations between the parasympathetic and sympathetic systems determine the basic variability of FHR [29,30,31,32].

The body’s nervous system causes the accelerations that constitute it as a result of the CNS action for fetal control movements. The action of the sympathetic system is essential for fetal survival and causes an increase in FHR. Conversely, the parasympathetic system decreases the fetal heart rate as a result of the stimulation of the carotid sinus chemoreceptors and of the aortic arch and peripherally located baroreceptors in the carotid bulb and aorta and centrally in the brain [29,30,31,32]. The stimulation of baroreceptors, induced by the pressure from the umbilical cord and the fetal head, contributes to a reduction in fetal heart rate (FHR), as evidenced by short-duration occurrences of variable decelerations [29,30,31,32].

Chemoreceptors are stimulated by the increase in hydrogen ions, the accumulation of CO_2_, and the decrease in the partial pressure of oxygen. The increase in the secretion of catecholamines is a reaction product in the upcoming fetal distress and manifests as a slowly evolving increase in FHR as a result of vasoconstriction and the redistribution of its flow blood to vital organs. The fetal stress response involves the release of catecholamines from the adrenal glands. The fetus’s capacity to withstand the stress induced by contractions and the pressure from the umbilical cord depends on its normal reserves, as well as on factors such as the presence of infection or meconium-stained amniotic fluid. The contractions of the myometrium during labor compress the fetal structures, including the umbilical cord, leading to significant alterations in the intrauterine environment. A reduced uteroplacental reserve is a consequence of pregnancies complicated by intrauterine growth retardation, prematurity, or pregnancy prolongation. The main goal of cardiotocography, especially in cases of shoulder dystocia, is to assess the degree of adequacy of the fetoplacental unit and the early detection of changes in the fetal heart rate indicative of fetal hypoxia, as well as the effectiveness of actions in preventing unwanted events depending on the timeliness of their implementation.

Hypoxemia (hypoxaemia) describes the condition in which there is a decrease in blood supply at the level of the placenta and umbilical cord and a decrease in oxygenation in the peripheral circulation of the fetus. This condition is often observed during normal childbirth; it is a result of uterine contractions, and most fetuses cope adequately for a long time with the occurrence of such episodes without establishing damage.

Hypoxia describes the situation where there is an interruption of blood supply for longer periods resulting in a decrease in the oxygenation of peripheral tissues and organs of the fetus. When hypoxia persists for more extended periods, the fetus activates the anaerobic metabolism that leads to the establishment of metabolic acidosis [29,30,31,32].

In fetuses with a reduced reserve such as intrauterine growth retardation, or prematurity or infection, the establishment of metabolic acidosis occurs earlier. The term asphyxia describes the extremely adverse situation in which there is a major worsening or prolonged interruption of reduced oxygenation leading to the inability to respond to compensatory mechanisms and the establishment of a combination of metabolic acidosis and hypoxia [29,30,31,32].

This leads to significant damage to vital organs such as the CNS and possibly intrauterine death. Long-term or chronic hypoxia is due to a reduction in placental blood flow for a long period of time associated with chronic pathological conditions such as pre-eclampsia or intrauterine growth retardation.

Before delivery, the fetus copes for a long time by redistributing blood flow to vital organs by slowing the rate of intrauterine growth and by controlling the increase in lactic acid levels. During delivery, this hypoxia is identified by a significant decrease in removing volatility and the emergence of repeated late slowdowns.

Gradually developing hypoxia is characterized by the abolition of the accelerations, the increase in the FHR, and the reduction in the variability over time. The decelerations become larger and of longer duration with a concomitant reduction in the duration of the intervals with the worsening of the hypoxia.

The presence of such a severity of hypoxic insult causes the fetal reaction expressed initially by:

1. the appearance of either variable decelerations (due to the compression of the umbilical cord) or the appearance of late decelerations (due to placental insufficiency);
2. the disappearance of accelerations (the result of an effort to conserve energy);
3. the gradual increase in FHR (due to hypoxia and the increase in catecholamine secretion);
4. the deepening and increase in the range of decelerations (with increasing hypoxia of the myocardium);
5. a progressive abolition of variability (due to a greater lack of oxygen resulting in autonomic nervous system depression) [29,30,31,32].

Acute hypoxia is characterized by a sudden decrease in placental/umbilical blood flow and develops over a short period (minutes). Causes include acute events such as placental abruption, hypertension, or uterine rupture. Recordings show prolonged decelerations progressing to bradycardia.

Immediate treatment is required by arranging the delivery or eliminating hypertonia. Subacute hypoxia is due to a recurrent compression of the umbilical cord. It worsens in cases of oligohydramnios or the prolongation of pregnancy, especially in the 20th stage of labor. The fact that hypoxic attacks have a slow progression at first and they are persistent after a sufficient period allows the gradual adaptation of the fetus.

These adaptive actions begin to lose their effectiveness when severe metabolic acidosis sets in (Ph ≤ 7) with the suppression of myocardial function, reduction in vascular tone, and establishment of hypotension, and ischemic brain damage. The pH in the fetal circulation decreases by 0.01–0.04 units/min. In general, the obstetrician typically has approximately 5 min, assuming the fetus is well-oxygenated, before the risk of severe hypoxia arises [2,33,34,35].

## 5. Clinical Manifestations of Dystocia

Dystocia, which, as we mentioned, is characterized by a slow progression with delayed rhythms or even pauses in the dilation of the cervix or the descent of the fetus, in practice, manifests itself in a prolongation of the latent phase, a slowing down or even a pause in the active phase, and with an extension of the extrusion stage. 

To fully understand the conditions related to dystocia, some introductory data for normal labor are necessary. 

### 5.1. Normal Labor

Normal labor is characterized by uterine contractions of progressively increasing intensity, frequency, and duration, which aim to efface and dilate the cervix and the descent and exit of the fetus

It is divided into three stages:

(a) The first stage or stage of expansion.
  This is divided into two sub-phases: the latent and the active.
  (1) The latent phase commences at the onset of labor, marked by the gradual effacement and initial dilation of the cervix, until it reaches a dilation of 3–4 cm. Based on new data, the diagnosis of the latent phase is based on the presence of contractions, cervical dilation < 6 cm on digital examination, slow cervical change, and clinician judgment. The upper time limits of this phase have been determined at 20 h for primiparous and 14 h for multiparous [7,18,19,20,21]. During this phase, the uterine contractions are of a relatively low intensity, short duration, and variable frequency.
  (2) The active phase ensues, culminating with the complete dilation of the cervix. In this phase, the contractions increase in intensity, frequency, and duration. The upper time limits have been set at 12 and 6 h for primiparous and multiparous, respectively [7,18,19,20,21]. At the same time, the minimum dilation rate limits have been established at 1.2 cm/h for primiparous and 1.5 cm/h for multiparous. Another parameter is the descent rate of the fetal head, which should be greater than 1 cm/h and 2 cm/h for primiparous and multiparous, respectively [7,18,19,20,21].
(b) The second stage or stage of extrusion

It begins with the full dilation of the cervix and ends with the exit of the fetus. The average duration of this stage is 50 min for the primiparous and 20 min for the multiparous. The upper time limits are considered to be 2 h for the first-born and 1 h for the first-born, added by 1 additional hour in case the pregnant woman has undergone regional anesthesia [7,18,19,20,21].

### 5.2. Shoulder Dystocia

Shoulder dystocia occurs when the biacromial diameter of the fetus is sufficiently large to impede or prevent the passage of the shoulders through the pelvic canal. Reported incidences of shoulder dystocia vary widely, with an estimated occurrence of approximately 1 in 150 (0.7%) of vaginal births. While there is a correlation between shoulder dystocia and fetal weight, fetal weight alone is not a reliable prognostic indicator for predicting its occurrence.

The frequency of shoulder dystocia ranges from 0.15% to 1.7%. However, many cases may go undiagnosed because preventive measures are often taken when its occurrence is considered possible. 

Among the risk factors for the manifestation of shoulder dystocia are all factors that predispose to a large or macrosomic fetus.

Shoulder dystocia is associated with increased perinatal morbidity and mortality. Injuries of the brachial plexus are not rare and occur during its stretching, from the excessive pulling of the fetal head in the attempt to exit the anterior shoulder. Clavicle fractures often accompany shoulder dystocia. It is thought that, when isolated, they have no particular clinical significance.

In prolonged dystocia, it is possible to observe fetal distress, suffocation, and death. Maternal complications of shoulder dystocia are postpartum hemorrhage from subsequent uterine atony, genital injuries, and postoperative infections in caesarean sections due to the reinsertion of the fetus’s head from the potentially septic environment of the vagina and perineum into the intrauterine cavity during the transabdominal delivery of the fetus. The treatment of shoulder dystocia involves a series of maneuvers aimed at releasing the shoulders and facilitating the delivery of the fetal trunk. Initially, gentle traction is applied to the fetal head while simultaneously exerting an extrusive effort from the placenta. If not already performed, a wide perineotomy is recommended. After clearing the newborn’s mouth and nose, gentle traction on the head is repeated while an assistant applies moderate suprapubic pressure.

This is followed by the McRoberts maneuver, with a strong flexion of the femur’s thighs toward the abdominal wall to straighten the sacrum and spine [21,36]. Most cases of shoulder dystocia are treated with these maneuvers. In a different case, Woods’ rotary maneuver is performed, to release the anterior shoulder, and, if this fails, the exit of the posterior shoulder of the fetus is attempted first [37].

Finally, as a last resort, inducing a fracture of the anterior clavicle or humerus and the Zavanelli maneuver with the reinsertion of the head into the pelvic canal and emergency caesarean section will assist in delivery [38]. Others have been described, which are not often applied today, such as the Rubin I and II manipulations and the manipulation proposed by Hibbard [26,39]. Symphysiotomy will only be performed in a case where all previous attempts have failed, with dubious results, as it is accompanied by significant maternal morbidity [40,41].

#### 5.2.1. Prolongation of the Latent Phase

We have an extension of the latent phase when its duration exceeds 20 h for primipara and 14 h for multipara. Causes include heavy sedation, regional anesthesia, immaturity of the cervix (low Bishop score), and false labor [6,9,16,17]. The latter refers to irregular contractions without a progressive increase in intensity, duration, and frequency. These contractions do not lead to the dilation of the cervix and are typically accompanied by discomfort in the lower abdomen. They are further weakened by the administration of sedative drugs.

After excluding the possibility of false labor, which reaches 10% of cases, the prolongation of the latent phase should be treated with rest and waiting, since 85% will automatically proceed to the active phase of labor, while the remaining 5% will require the administration of oxytocin [42,43,44]. Amniotomy should be avoided due to the possibility of false labor. Certainly, if there are indications necessitating the delivery of fetus, such as the prolonged rupture of fetal membranes or abnormal fetal heart rate patterns, waiting should be replaced by expediting delivery, with caesarean section being considered if other methods fail.

#### 5.2.2. Prolongation or Slowing Down of the Active Phase

When the active phase exceeds 12 h for the primipara and 6 h for the multipara, it is considered prolonged. We have a slowing of the active phase when the rate of cervical dilation is less than 1.2 cm/h in primiparous and 1.5 cm/h in multiparous, or the rate of descent of the fetal head is less than 1 cm/h and 2 cm/h for primigravida and multigravida, respectively.

The causes include heavy sedation, regional anesthesia, and abnormal projections of the fetus [31,32]. 

After ruling out disproportion and ensuring there are adequate uterine contractions, it is advisable to adopt a wait-and-see approach. In cases of sluggish myometrial activity, interventions such as amniotomy and the initiation of oxytocin administration are recommended. If cervical dilation fails to progress by at least 2 cm within the next 4 h despite these measures, caesarean section is indicated.

#### 5.2.3. Pause of the Active Phase

The Cessation of cervical dilatation for at least 2 h or the cessation of fetal descent for 1 h is characterized as a pause in the active phase of labor. Newer trends support increasing the follow-up time limit, before the diagnosis of cessation of dilatation is made, from 2 to 4 h [43,44]. The causes include the relative disproportion due to the abnormal presentation or position of the fetus, with the possible secondary exhaustion of the myometrium.

If the latter case is ruled out, the solution proposed is the reinforcement of contractions with oxytocin and/or amniotomy. If cervical dilation does not increase by 2 cm within the next 4 h, caesarean section is indicated [43,44].

#### 5.2.4. Extension of the Expulsion Stage

During the second stage of labor, if it extends beyond 2 h for primiparous women or 1 h for multiparous women, with an additional hour permitted in the case of regional anesthesia administration, this delineates the prolongation of the expulsion stage [7,25,26]. The causes include the disproportion and inadequacy of the forces exerted by the uterus and the placenta. As the dilation of the cervix is completed, the placenta feels the need to push out.

Myometrial contractions are enhanced by the administration of oxytocin, after absolute disproportion has previously been ruled out, while further management includes fetal ulceration using a suction or metallic embryonator, if the height of the projecting degree is below the +2 point and the caesarean section, in case it is above it.

In the context of dystocia, in the sense of pathological labor, the case of rapid progression of labor, called acute labor, is often considered.

#### 5.2.5. Precipitous Labor

It is characterized by rapid development and termination within 3 h. The frequency is approximately 2% [23]. The related term precipitous labor includes those cases where the cervical dilation rate is at least 5 cm/h for primiparous and 10 cm/h for multiparous. The complications of acute labor concern both the mother and the fetus. Pre-eclampsia may present with the rupture of the uterus and general injuries of the pelvic canal, embolism of amniotic fluid, and uterine weakness after hysterectomy.

The newborn can manifest hypoxygenemia, due to the frequent and intense contractions of the uterus, which obstruct the feto-placental circulation. Intracranial and brachial plexus injuries have been associated with acute delivery, while the neonate may suffer injuries from a fall to the ground if delivery occurs in an inappropriate location without obstetric monitoring. In this case, the lack of personnel qualified to perform resuscitation of the newborn should also be taken into account, if this is required [7,25,26].

Various ways of slowing down and controlling acute labor have been attempted, such as the administration of analgesics and tocolytics and general anesthesia with halothane or isoflurane, but with dubious results. What should not be omitted is the immediate discontinuation of oxytocin, if it is administered. Given that a history of acute labor raises the probability of recurrence in subsequent pregnancies, it would be advisable to plan for the induction of labor and placental delivery shortly before the anticipated delivery date.

## 6. Prevention—Prediction

Several risk factors implicated in the pathogenesis of shoulder dystocia may arise before or during normal vaginal delivery, thereby increasing the likelihood of its occurrence. However, these factors alone are insufficient to reliably predict dystocia, as there are instances of dystocia occurring without the presence of these risk factors. Furthermore, most cases of dystocia cannot be anticipated or prevented due to the lack of accurate methods for identifying embryos at risk of developing this complication [19,45,46,47].

The main risk factors that can appear before delivery are fetal macrosomia, gestational or pre-existing diabetes mellitus, and a history of shoulder dystocia in a previous delivery. Risk factors that occur during labor are the administration of oxytocin, prolonged first and second stages of labor, and invasive vaginal delivery using a suction or metallic embryo tube [19,45,46,47].

Other minor risk factors are a history of a macrosomic fetus in a previous pregnancy, maternal short stature, maternal obesity, pelvic anomalies, multiparity, and prolonged gestation. It is understood that efforts are being made to improve some of the risk factors that appear during pregnancy so that there is a better prognosis and management of childbirth, despite the great difficulty in predicting shoulder dystocia [19,45,46,47].

If there is a clinical suspicion of macrosomia, performing an ultrasound examination of fetal development can help to make important clinical and interventional decisions; however, it has limited preventive and diagnostic accuracy. In cases of gestational diabetes mellitus, in order to prevent macrosomia, it is advisable to closely manage diabetes through the adherence to a diabetic diet, regular monitoring of glucose levels, and administration of insulin by an endocrinologist if deemed necessary. Infants born to diabetic mothers face a two- to four-fold higher risk of experiencing shoulder dystocia compared to infants born to non-diabetic mothers of similar weight. In overweight or obese women (BMI > 25), physical exercise combined with an appropriate dietary treatment is recommended to reduce the possibility of a macrosomic fetus. During pregnancy, the recommended weight gain is 11.5 kg to 16 kg for women with a normal body mass index [19,45,46,47].

In the general population, physical exercise is recommended before or during pregnancy in order to limit the possibility of gestational diabetes, macrosomia of the fetus, and excessive weight gain during pregnancy.

To achieve a better prevention of shoulder dystocia, the induction of labor, as well as caesarean section, can be applied. The elective induction of labor based on suspected macrosomia is not a reasonable strategy and does not improve perinatal outcome [48,49,50,51].

The elective induction of labor may be considered for women diagnosed with gestational diabetes mellitus and suspected macrosomia, provided they have reached a gestational age exceeding 39 weeks and possess an appropriately prepared cervix. Elective caesarean section may be warranted when the estimated fetal weight exceeds 4500 g and is coupled with maternal gestational diabetes, or when the estimated fetal weight surpasses 5000 g without concurrent gestational diabetes [48,49,50,51].

During labor, in the case of macrosomia of the fetus and the impossibility of the progression of the second stage of labor (expulsion), assisted vaginal birth should be avoided and caesarean section should be performed. Elective caesarean section should be recommended in the case of a history of shoulder dystocia in a previous birth, as the rate of recurrence of dystocia in subsequent births is 10 times higher than in the general population.

## 7. Effects

The complications of shoulder dystocia are divided into maternal and neonatal. The increase in maternal morbidity is mainly due to postpartum bleeding that can come from uterine atony, cervical tears, and vaginal and perineal tears. The dissection of the symphysis pubis and rupture of the uterus are less commonly recorded. The incidence of the above complications does not appear to be affected by the number or type of manipulations applied to treat shoulder dystocia, except for symphysis pubis dissection and transient uterine neuralgia associated with the McRoberts manipulation mentioned below [51,52,53,54,55].

Neonatal morbidity is usually associated with fetal injury occurring in 17% to 25%, with brachial plexus paresis being the most common. This complication arises usually in 7% to 20% of fetuses with shoulder dystocia. Usually, lesions of the right brachial plexus are more common than the left, probably because of the more frequent left anterior occipital position of the fetus.

Brachial plexus paresis is classified into Erb’s paresis, Klumpke’s paresis, and mixed brachial plexus palsies. Erb’s paresis is the most common and involves the A5 to A6 nerve roots. Klumpke’s paresis is rarer and involves nerve roots A8 to T1. Mixed paresis involving nerve roots A5 to T1 are ranked last [56,57,58,59].

Most brachial plexus injuries resolve during the neonatal period, usually within six to twelve months, with no or minimal neurological deficit. However, 10% of cases of Erb’s palsy never resolve. Further neonatal injuries associated with neonatal morbidity in shoulder dystocia include clavicle fracture and humerus fracture [56,57,58,59].

Clavicle fractures typically heal without complications, although some cases may involve an associated injury to the lungs and underlying vessels. Humerus fractures typically heal without resulting deformities [56,57,58,59].

In addition to neonatal trauma, additional complications associated with neonatal morbidity in shoulder dystocia are fetal hypoxia, ischemic encephalopathy, and even neonatal death. The latter complications are because, after the delivery of the head, the umbilical cord is pressed between the body of the fetus and the maternal pelvis

Thus, the rapid recognition and decisive treatment of shoulder dystocia is necessary to avoid permanent neonatal damage [56,57,58,59].

## 8. Management 

A variety of dystocia manipulations have been described to free the anterior shoulder from its wedged position behind the maternal symphysis pubis.

The purpose of these manipulations is to increase the size of the maternal pelvis, reduce the biacromial diameter, and change the biacromial diameter inside the pelvis.

In women with risk factors, the obstetrician should use the ‘shoulder delivery immediately after head exit’ maneuver to allow the fetus to be delivered without interruption until the anterior shoulder exits.

In case of entrapment, the initial treatment will include the slight downward traction of the fetal head alongside the pushing efforts of the mother. The bending, twisting, and sudden movement of the neck should be avoided to avoid damaging the fetal brachial plexus.

Pressure on the bottom of the uterus is contraindicated, as it does not offer any help and, instead, increases the wedging with the risk of injury to the fetus and the mother [59,60,61].

The need for a perineotomy should always be considered and evaluated. A wide perineotomy does not directly solve the problem because the soft tissues are not involved, but it provides the necessary space for performing intravaginal manipulations. 

### 8.1. Initial Treatments for Shoulder Dystocia

(a) McRoberts maneuver

The McRoberts maneuver is one of the first and simplest steps in treating shoulder dystocia. To perform the maneuver, the thighs are bent with a little abduction so that they touch the abdomen of the patient. In this way, an increase in the anteroposterior diameter of the pelvis is achieved as well as a straightening of the lumbar lordosis of the spine (pronouncement of the obstetrician); 40% of shoulder dystocia are successfully treated with the McRoberts maneuver [62,63,64].

(b) Suprapubic pressure

The suprapubic pressure maneuver is a fundamental aspect of managing shoulder dystocia. This technique involves applying pressure above the pubic bone on the anterior shoulder of the fetus, aiming to adduct the shoulders. Simultaneously, gentle traction is continued by the obstetrician. Initially, the pressure should be continuous and then transition to a rhythmic pattern, similar to cardiopulmonary resuscitation. When combined with the McRoberts maneuver, this approach successfully resolves shoulder dystocia in over 50% of cases.

(c) Intravaginal Maneuver

In addition to the primary maneuver described above, there are also intravaginal techniques available for managing shoulder dystocia. These include the Rubin manipulation, the Woods Screw maneuver, the reverse Woods Screw maneuver, and the maneuver for displacing the posterior upper limb from the birth canal. During the Rubin maneuver, the obstetrician’s hand is inserted behind the anterior shoulder, applying pressure in the direction of the fetal chest.

(d) The Woods Screw maneuver

The technique, first described by C.E. Woods in 1943, is an intravaginal manipulation performed concurrently with the Rubin maneuver. In this maneuver, the obstetrician’s second hand approaches the posterior shoulder from the anterior surface of the fetus, reinforcing the effort to rotate the shoulder in the same direction as the Rubin manipulation.

Another maneuver involves placing the obstetrician’s hands behind the posterior shoulder and in front of the anterior shoulder, attempting to rotate the fetus in the opposite direction to the previous manipulations (reverse Woods Screw maneuver).

Another intravaginal maneuver is the movement of the posterior upper limb into the genital canal to reduce the diameter of the shoulders. To perform this maneuver, the obstetrician inserts his hand into the vagina trying to reach the posterior upper end of the fetus. After the forearm is reached, the elbow is bent so that the forearm comes out in front of the fetal chest. The traction should not be sharp to avoid fracturing the upper extremity.

In this way, the posterior upper extremity exits, and, thus, the anterior shoulder descends lower than the pubic symphysis facilitating delivery.

(e) The knee–elbow position

The maneuver of the knee–elbow position is effective, safe, and fast.

During this maneuver, it is crucial that we position the mother in the knee–elbow position. This position facilitates an increase in pelvic diameters: the strait is augmented by approximately 10 mm, and the transverse measurement of the pelvic outlet can increase by up to 20 mm.

During rotation and with the help of gravity, the anterior fetal shoulder is often released. It is important, in this case, to pull the fetus downwards, trying to achieve the delivery of the posterior shoulder.

(f) Clavicle fracture

An alternative way of dealing with shoulder dystocia is a clavicle fracture.

During this manipulation, upward pressure is applied to the middle of the clavicle to prevent damage to the subclavian vessels.

### 8.2. Last-Resort Treatments for Shoulder Dystocia

(a) Zavanelli procedure

Ultimately, the last-resort procedures for managing shoulder dystocia encompass the Zavanelli procedure, abdominal surgery with hysterotomy, and symphysiotomy. During the Zavanelli maneuver, efforts are made to reposition the fetal head, followed by an emergency caesarean section. The fetal head is gently pushed back against the delivery mechanism, and pressure is sustained until the caesarean section can be performed. Tocolysis is typically administered before commencing the procedure.

During abdominal surgery with hysterotomy, vaginal delivery is facilitated, as the fetus is rotated as in the intra-abdominal Woods Screw maneuver. Symphysiotomy is conducted under local anesthesia following bladder catheterization. This is followed by the separation of the fibrocartilage of the pubic symphysis. Given that the procedure requires at least two minutes from the decision-making moment, it is essential to initiate it within five to six minutes following the birth of the infant’s head.

(b) Symphysiotomy

Symphysiotomy is conducted under local anesthesia following bladder catheterization. This is followed by the separation of the fibrocartilage of the pubic symphysis. Given that the procedure requires at least two minutes from the decision-making moment, it is essential to initiate it within five to six minutes following the birth of the infant’s head. Symphysiotomy should be used only when other manipulations have failed and caesarean section is not possible [62,63,64].

### 8.3. Overall Management 

The early diagnosis and immediate treatment of shoulder dystocia lead to a better maternal and neonatal outcome. Research data indicate that the rate of fetal hypoxia and death is reduced if the time between the delivery of the head and body is less than 5 min. Once shoulder dystocia is diagnosed, it is crucial to refrain from applying any additional traction, apart from the standard gentle traction required for shoulder delivery in normal vaginal births. Excessive traction along the axis of the fetal spine should be avoided, as it may result in serious injuries to the mother and severe neurological damage to the newborn.

The attending physician or midwife should discontinue any extrusive labor efforts exacerbating shoulder impingement, while simultaneously alleviating fundal pressure, as it can heighten the risk of vaginal complications and uterine rupture. Consequently, further manipulations during delivery should be conducted by the practitioner with the assistance of specialized and knowledgeable personnel.

According to the American Academy of Family Physicians (AAFP) and the obstetric emergency management and treatment program (ALSO), the HELPERR algorithm is proposed as an important clinical tool for shoulder dystocia.

All technical steps are equally important and must be carried out adequately; a time of 30–60 s is recommended for each manipulation:

Help, Evaluate (for episiotomy), Legs (McRoberts’ position), Pressure (suprapubic), Enter (rotational maneuvers), Remove (posterior arm), and Roll (hands and knees).

Upon immediate recognition of dystocia, it is imperative to summon additional personnel for the delivery, including extra midwives, and nursing staff, as well as specialists in resuscitation and postnatal care. It is essential to ensure the presence of an anesthesiologist. The availability of staff may vary depending on the Health Unit where the delivery occurs. Furthermore, it is advisable to conduct the annual participation of maternity staff in specialized seminars to facilitate their continuous training and updates. Despite the extensive research conducted on perineotomy, there is no evidence supporting its efficacy for the prevention and management of shoulder dystocia. The perineotomy application alone does not resolve the problem. However, it can be beneficial in situations where space is limited. For instance, when there is insufficient room in the vagina for the obstetrician’s hand, a perineotomy can facilitate access for intravaginal manipulations.

As perineotomy is quite difficult after the delivery of the head and can cause injury to the newborn, the obstetrician should evaluate its utility and resort to it before delivery if dystocia is suspected. Finally, perineotomy has been blamed for increased rates of severe perineal tears.

McRobert’s maneuver is often the initial step in addressing shoulder dystocia, as it is relatively simple and less invasive compared to other manipulations. This maneuver involves bending and abducting the thighs of the mother so that they come into contact with her anterior abdominal wall. This action straightens the lumbar spine and increases the anterior–posterior diameter of the pelvis. During the maneuver, the bed should be completely flat, with all pillows beneath it removed, and assistance from the staff on either side of the bed is necessary to bend the thighs appropriately. Gentle traction is then applied to facilitate the delivery of the anterior shoulder.

This maneuver is effective in up to 90% of cases, with better results if combined with suprapubic pressure. If the delivery of the fetus does not occur in 30–60 s, a subsequent manipulation is attempted.

Suprapubic pressure (Rubin maneuver 1) is applied by an assistant, on the mother’s side, whose hands are placed on the anterior shoulder. Its purpose is to reduce the biacromic diameter, with the palmar surface of the assistant’s hand down and to the side, on the posterior surface of the anterior shoulder, so that it can be released and pass through the pubic symphysis

It can be applied alongside the McRoberts maneuver while the obstetrician applies gentle traction. Pressure is applied as in CPR, and there is no clear difference in the effectiveness of continuous and rhythmic pressure. If the method fails, in 30–60 s, a further operation should follow.

Intravaginal maneuvers attempt to rotate the fetus so that the anterior pubic symphysis is released and passed. The Rubin II maneuver is performed by inserting the obstetrician’s fingers behind the anterior shoulder, and applying rotational pressure to the fetal chest wall.

It can also be applied alongside the McRoberts manipulation. If this fails, it can be combined with the Wood maneuver, in which the obstetrician uses the fingers of the other hand, with which he applies rotational pressure in the same direction as that of Rubin I, on the anterior surface of the posterior shoulder. A perineotomy is deemed necessary for this operation. If both of these manipulations fail, the reverse Wood manipulation may be followed, in which rotational pressure is applied with the fingers behind the posterior shoulder to rotate it into the oblique plane [63,64,65]. After the shoulder rotation maneuvers prove ineffective, the next step involves attempting delivery of the entire posterior upper limb. This is achieved by applying traction and flexion to the posterior elbow, bringing it in front of the fetal chest, followed by the traction and delivery of the posterior upper limb from the hand up to the shoulder. This technique facilitates the delivery of the fetus. However, it is important to note that this particular method is associated with humerus fractures, with a reported frequency ranging from 2% to 12% [56,57,66,67,68,69].

The knee–elbow position of the epitome is effective, with a success rate of up to 83% in cases of dystocia. Depending on the circumstances, the obstetrician will attempt to turn the patient into this position either before or after attempting the above manipulations. In this position, delivery can be carried out either by gentle downward traction, in which case the delivery of the posterior shoulder occurs first, or by intravaginal manipulations to follow [25,38,70,71].

This position is suitable when the patient is thin and not under epidural anesthesia. The same approach may not be suitable in the opposite scenario, such as when the patient has a Foley urinary catheter or is being monitored with a cardiotocogram. In such cases, intra-abdominal manipulations are typically preferred.

If all the previously described maneuvers fail, last-resort maneuvers are pursued, which must be approached with utmost caution before resorting to them, to mitigate maternal morbidity and mortality, particularly in the hands of inexperienced practitioners. One such manipulation is the intentional fracture of the fetus’s clavicle, aimed at reducing shoulder resistance [25,34,38,70,71,72].

The prognosis for shoulder dystocia cases has shown improvement following specialized seminars for maternity ward staff. Studies have indicated a decrease in the rate of neonatal trauma from 9.3% before these training seminars to 2.3% after their implementation. Some studies emphasize the importance of conducting such training sessions on an annual basis. Ultimately, it is recommended to establish a shoulder dystocia management protocol in every clinic to ensure the organized and timely treatment [25,34,38,70,71,72].

## 9. Conclusions

Shoulder dystocia is the nightmare of obstetricians. It is a complication of normal vaginal delivery, which, despite its low frequency, can have unpleasant consequences for both the mother and the newborn.

While there are risk factors that have been associated with the onset of dystocia, it remains extremely difficult to predict.

Several maneuvers have been described that can help deal with this dramatic situation. Various protocols and algorithms have been drawn up by groups of scientists in order to organize the actions of the obstetrics team in a shoulder dystocia emergency.

In such cases, crucial attributes include speed and determination. Therefore, the obstetric team tasked with managing such emergencies should be scientifically qualified and adequately trained, preferably through simulation scenarios.

At the same time, however, the necessity of the thorough theoretical and practical training of the new obstetrician in the delivery room should be emphasized, so that he is able to recognize in time the signs that indicate the appearance of dystocia, before it leads to obstetrical impasses and hasty actions, which “impose” risks for the mother and the fetus, for situations that should have been foreseen.

Seminars featuring simulated scenarios offer valuable training opportunities for obstetricians and midwives, enhancing their proficiency in handling immediate danger situations effectively.

## Data Availability

No new data were created or analyzed in this study. Data sharing is not applicable to this article.
